# Distance-dependent connectivity in the brain facilitates high dynamical and structural complexity

**DOI:** 10.1007/s11571-025-10398-9

**Published:** 2025-12-26

**Authors:** Victor J. Barranca

**Affiliations:** Department of Mathematics and Statistics, Swarthmore College, 500 College Avenue, Swarthmore, PA 19081 USA

**Keywords:** Neuronal networks, Nonlinear dynamics, Complexity, Information theory, Connectomics

## Abstract

Recent experiments have revealed that the inter-regional connectivity of the cerebral cortex exhibits strengths spanning over several orders of magnitude and decaying with distance. We demonstrate this to be a fundamental organizing feature that fosters high complexity in both connectivity structure and network dynamics, achieving an advantageous balance between integration and differentiation of information. This is verified through analysis of a multi-scale neuronal network model with nonlinear integrate-and-fire dynamics, incorporating inter-regional connection strengths decaying exponentially with spatial separation at the macroscale as well as small-world local connectivity at the microscale. Through numerical simulation and optimization over the model parameterspace, we show that inter-regional connectivity over intermediate spatial scales naturally facilitates maximally heterogeneous connection strengths, agreeing well with experimental measurements. In addition, we formulate complementary notions of structural and dynamical complexity, which are computationally feasible to calculate for large multi-scale networks, and we show that high complexity manifests for each over a similar parameter regime. We expect this work may help explain the link between distance-dependence in brain connectivity and the richness of neuronal network dynamics in achieving robust brain computations and effective information processing.

## Introduction

Characterizing the structure-function relationship in neuronal networks is crucial to elucidating the nature of information processing in the brain. Both experimental and theoretical studies have increasingly shown that the connectivity both within and between brain regions is fine-tuned to optimally perform specific network functions (Sporns 2013; Mountcastle 1997; Rosa and Tweedale 2005; Friston 2009; Colby and Duhamel 1991; Morel et al. 1993; Albright et al. 1984; Treisman 1996; Damoiseaux and Greicius 2009). The structural profile of neuronal networks is generally neither completely ordered nor fully random (Bartfeld and Grinvald 1992; Weliky et al. 1996; Rubinov and Sporns 2010; Barranca and Zhou 2019), facilitating a rich repertoire of neuronal network dynamics necessary for proper brain function (Albert and Barabási 2002; Massimini et al. 2005; Petri et al. 2014; Liu et al. 2008; Netoff et al. 2004; Gray et al. 1989; Nematzadeh et al. 2014). A key theoretical question that often arises, however, is why certain patterns of connectivity are so pervasive and what beneficial effects might they impart on neuronal network computation?

Experimental studies strongly indicate that neuronal connectivity, both locally and globally, is profoundly impacted by the spatial separation between neurons and typically decreases in strength with distance (Brunel and Sergi 1998; Song et al. 2005; Wang et al. 2012; Tononi et al. 1998; Barranca et al. 2015). While *distance-dependent connectivity* promotes short network wiring-lengths and efficient energy consumption, the resultant network dynamics must still be robust enough to carry out a diversity of brain functions and persist across environmental pressures (Kirschner and Gerhart 1998; Bassett et al. 2010; Lennie 2003; Niven and Laughlin 2008; Chen et al. 2006), [31]. In addition to obeying an exponential distance rule, inter-regional connectivity in the macaque monkey cortex spans several orders of magnitude (Markov et al. 2013a, b), though whether this arises as a natural consequence of distance-dependence or for a distinct functional purpose remains unknown and is a subject of investigation in this work. Human and mouse brains also appear to demonstrate connectivity strengths that decay with physical distance (Perinelli et al. 2019; Song et al. 2014; Trinkle et al. 2021; Rubinov et al. 2015), but more detailed and large-scale connectome data is still necessary to fully characterize the strength distribution of connections in humans (Sepulcre et al. 2010).

The information-theoretic notion of *complexity* is widely used to characterize the dynamical richness of interactions between functional modules of neurons in the brain, measuring simultaneously the degree of both integration and differentiation of information (Tononi et al. 1994; Barnett et al. 2009; Barranca et al. 2015). Under the assumption that neuronal interactions are approximately statistically stationary over a time-scale of interest and well described by a linear multivariate stochastic process with Gaussian dynamics, the notion of complexity may be re-framed as a structural rather than dynamical construct, requiring only knowledge of network connectivity. This framework of complexity, combined with other tools of graph theory, has been utilized to study mental health disorders, consciousness, as well as brain imaging (Stam 2005; Tononi and Edelman 1998; Garrity et al. 2007; Whitfield-Gabrieli et al. 2009). In this work, we hypothesize that distance-dependent connectivity among areas of the cerebral cortex facilitates high complexity in both network structure and dynamics, thereby emerging as a ubiquitous organizing feature.

Using a multi-scale network model incorporating distance-dependent connection-strength among cortical regions, we first demonstrate that this ubiquitous structure does indeed increase structural complexity and naturally encourages variability in inter-regional connection strengths, as observed experimentally. Taking into account individual neuronal dynamics and local small-world connectivity in each region, we present a computationally efficient framework for describing the dynamical complexity corresponding to a given network configuration. We show that in the dynamical regime of maximal complexity, the network activity is approximately Gaussian, demonstrating a combination of both synchrony and uncorrelated activity among cortical regions. Unlike previous studies, in prescribing the microscale dynamics of our model system, we use a spiking model for each neuron via non-linear integrate-and-fire (I&F) dynamics (Barranca et al. 2014; Mirollo and Strogatz 1990; Peskin 1975). While we focus on a specific mechanistic model of the cerebral cortex, the presented framework for characterizing multiscale network connectivity and dynamics is fully generalizable to other network models with nonlinear dynamics, which we expect to prove useful for future studies in theoretical neuroscience and network science in general.

The structure of the paper is as follows. In Sect. 2.1, we introduce the connectivity and dynamics of our multiscale model of the cerebral cortex. Then, we briefly introduce the framework of information-theoretic complexity in Sect. 2.2. Based on the inter-regional connectivity of our model, we study in Sect. 3.1 the network structural complexity as a function of distance-dependence in connection-strength and demonstrate that maximal structural complexity arises from connectedness spanning intermediate spatial scales. We further show that this maximal structural complexity naturally gives rise to highly-variable connection-strengths that range over several orders of magnitude. Next, in Sect. 3.3, we introduce a new multi-scale notion of dynamical complexity. We demonstrate that the network model exhibits high dynamical complexity in the region of maximal structural complexity, which corresponds to a mixture of both synchronous and irregular activity among regions as well as approximately Gaussian dynamics. Finally, we summarize our findings and discuss their implications in Sect. 4.

## Methods

### Network model structure and dynamics

To study the dynamical and structural effects of experimentally observed distance-dependent connectivity in cortical networks, we construct a mechanistic network model incorporating regional structure and nonlinear individual neuronal dynamics. In formulating the large-scale connectivity, we take into account recent work demonstrating that inter-regional connection strength in the macaque cerebral cortex decreases exponentially with distance (Markov et al. 2013a, b). To introduce spatial structure, we partition our model into cortical regions arranged on a grid lattice. For any set of two neurons in different regions, *i* and *j*, the probability of a connection is

1 $$\begin{aligned} P(i,j)= \rho \exp (-[(x_i-x_j)^2 +(y_i - y_j)^2]/[2\sigma ^2] ), \end{aligned}$$

where $(x_i,y_i)$ are the coordinates of the $i^{th}$ region and $(x_j,y_j)$ are the coordinates of the $j^{th}$ region. Parameter $\sigma$ determines the expected distance over which neurons in differing regions are connected and scaling factor $\rho$ determines the regional connection density. We note that the connection probability for each distinct pair of neurons is computed separately, such that the probability of a specific inter-regional connection between a pair of neurons is independent of all other connections.

On the finer scale of connectivity among neurons in a single region, we use a small-world network framework to describe the local structure. Small-world networks simultaneously demonstrate low average path-lengths and high clustering among nodes, thereby facilitating rapid and low-cost communication, and they are therefore prevalent in both natural and man-made systems (Watts and Strogatz 1998; Amaral et al. 2000; Latora and Marchiori 2001; Barranca et al. 2015). With respect to neuronal networks in particular, small-world structure has been observed on both small and large scales, and thus we choose to model connectivity within each region accordingly (Heuvel et al. 2008; Sporns and Honey 2006; Bassett and Bullmore 2006; Varshney et al. 2011; Barranca et al. 2019).

For each regional network of *m* nodes, we construct a small-world network by randomly perturbing the connectivity of a regular ring lattice in which each node is initially connected to its nearest neighbors with connection density *c*. With probability *p*, we randomly rewire each existing connection in the network, removing the initial connection and randomly forming a new connection with equal probability among all possible nodes. We remark that for approximately $0.01< p < 0.1$, there are sufficiently many rewired connections such that the network is typically considered small-world (Watts and Strogatz 1998). In this case, rewired edges tend to link clusters of highly-connected nodes, decreasing the average path length while maintaining a critical mass of clustered connections that remain from the original ring lattice. Since local cortical circuits are generally sparsely connected, as are small-world networks by definition, we assume that the connection density in each region is $c=0.1$ and also choose rewiring probability $p=0.1$ to achieve sparse small-world local connectivity (Markram et al. 1997; Mason et al. 1991; He et al. 2007; Achard and Bullmore 2007; Barranca et al. 2019).

To reflect the multi-regional nature of the full network model, we assume there are *z* regions, such that $Y_g$ is the set of indices for neurons in the $g^{th}$ region and there are $n=zm$ total neurons in the model. The recurrent connectivity among all neurons in the model is prescribed by $n \times n$ matrix $A=(A_{ik})$, and we define the strength of an existing incoming connection into the $i^\textrm{th}$ neuron from the $k^\textrm{th}$ neuron as

2 $$\begin{aligned} A_{ik}= {\left\{ \begin{array}{ll} 1/m, & \text{ if } k \in Y_g \\ 1/{N_{in}(i)}, & \text{ if } k \notin Y_g \end{array}\right. }, \end{aligned}$$

where *m* is the total number of neurons in a region, $i \in Y_g$, and $N_{in}(i)$ is the total number of connections incoming into the $i^\textrm{th}$ neuron. If a pair of neurons is not connected, as determined by the local (small-world constructed) and inter-regional (distance-dependent) connectivity, then the corresponding connection strength $A_{ik}=0$. Therefore, if two neurons are connected in the same region, their connection strength is 1/*m*, normalized by the number of neurons in the region. In the case that neurons of different regions are connected, their connection strength is $1/{N_{in}(i)}$, normalized by the number of incoming connections received by the $i^\textrm{th}$ neuron. This inter-regional connection-strength normalization reflects that on average the total input into each neuron is approximately the same through synaptic scaling, such that neurons with many in-links will weight each input less heavily than neurons with fewer incoming connections (Turrigiano 2007; Rabinowitch and Segev 2008; Goel et al. 2006; Davis 2006; Barnett et al. 2009; Tononi et al. 1994).

In Sect. 3.3, we analyze the dynamical complexity of our model network under the assumption of nonlinear I&F neuronal dynamics. In this case, we use a conductance-based neuronal model such that the membrane-potential dynamics of the $i^\textrm{th}$ neuron, $v_i$, are determined by the differential equation

3 $$\begin{aligned}&\tau \frac{dv_i}{dt} = -(v_i- V_R) + f_i\sum _{r} G(t,t_{i,r},\tau _E)(v_i-V_E) \nonumber \\&+ \sum _{\begin{array}{c} k=1 \\ k\ne i \end{array}}^n \sum _{l} A_{ik} G(t,\tilde{t}_{k,l},\tau _S)(v_i-V_E) , \end{aligned}$$

until reaching a sufficiently high value, $V_T=1$, at which point the membrane potential is instantaneously reset to resting voltage, $V_R=0$. At this instant, the neuron undergoes an action potential (fires or spikes) and affects the conductance of neighboring neurons that are post-connected to it. The conductance changes are prescribed by the alpha function

4 $$\begin{aligned} G(t,\tilde{t}_{k,l},\tau _S)= {\left\{ \begin{array}{ll} \left( \frac{t-\tilde{t}_{k,l}}{\tau _S^2}\right) \exp (-(t-\tilde{t}_{k,l})/\tau _S), & \text{ if } t \ge \tilde{t}_{k,l}, \\ 0, & \text{ if } t < \tilde{t}_{k,l} \end{array}\right. } \end{aligned}$$

where $\tilde{t}_{k,l}$ denotes the $l^\textrm{th}$ time that the $k^\textrm{th}$ neuron fires. The model is nondimensionalized with a time-scale of $\tau = 20$ms, equivalent to a conductance of $50s^{-1}$, synaptic conductance decay time-scale $\tau _S=1$ms, and excitatory reversal potential $V_E=3$, which is in the parameter regime typically produced in votlage normalization assuming a resting membrane potential of $-70mV$ and AMPA-type post-synaptic currents (McCormick et al. 1985; Kovačič et al. 2009; Shelley et al. 2002).

We assume neurons in each region are externally driven by a Poisson spike-train emanating from a large set of firing neurons outside of the model network, akin to AMPA-type excitatory post-synaptic potentials (EPSPs), reflecting the realistic scenario in which a given neuron is subject to a large number of spikes arriving independently at random times (Werner and Mountcastle 1965; Bair et al. 1994; Barranca et al. 2014). The external EPSPs have time-constant $\tau _{E}=2.728$ms and strength $f_{E}=0.1$, arriving into the $i^\textrm{th}$ neuron at times prescribed by $t_{i,r}$ with rate $\nu _{E}=500$ incoming spikes per second (Brown and Johnston 1983; Koch 1999; Rangan and Cai 2007). With this choice of parameters, the neuronal firing rates in an isolated region are moderate and the network dynamics are primarily in the fluctuation-driven dynamical regime in which firing events are largely caused by irregular temporal fluctuations in the voltage, as often found in diverse experimental settings and theoretical analyses (Cai et al. 2004; Anderson et al. 2000; Barranca 2023). The results of this work are not sensitive to perturbations in the Poisson input rate so long as the network remains roughly in the same dynamical regime. Similarly, the specific choice of rewiring probability, $p= 0.1$ is not crucial, as similar results are obtained across the small-world parameter regime of $0.01< p < 0.1$. Since it is reasonable to assume that the incoming input strength across cortical regions may be variable, in each region we multiply the external EPSP strength by a uniformly randomly chosen weight factor, $w_g$, such that the total external EPSP strength for the $i^\textrm{th}$ neuron in region *g* is $f_i=w_g f_{E}$, where $0.8 \le w_g \le 1.2$. Unless specified otherwise, we will assume that each region contains $m=100$ neurons and there are $z=9$ cortical regions arranged on a $3 \times 3$ square lattice.

**Fig. 1:**
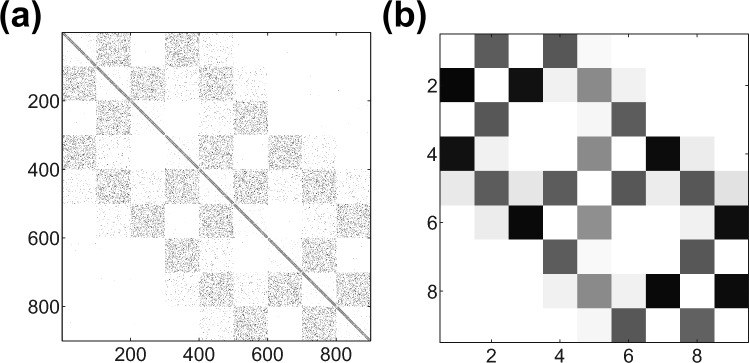
Model network connectivity. **a** Connectivity matrix, *A*, for a full neuronal network model with $z=9$ regions each containing $m=100$ neurons. **b** Macro-scale connectivity matrix, *R*, depicting the connection strength between the $z=9$ regions. In each case, darker colors indicate stronger connectivity. For this example network, the parameter choice corresponding to the distance-dependence indicated by Eq. (1) is $\rho =0.9$ and $\sigma = 0.5$. Regional locations on the grid lattice are indexed from left to right in top-down order

We depict in Fig. 1 the connectivity for an example model network realization, showing the full connectivity matrix for the network in panel (a). Locally, each region ($m \times m$ blocks along the main diagonal) displays small-world connectivity, marked by a moderate number of rewired connections between clusters of neurons, and we see distance-dependent connectivity on a global level. In Fig. 1b, for visualization purposes, we plot the *macroscale connectivity matrix*, *R*, representing the connection strengths among the $z=9$ regions in the cortical model. In particular, to compute the strength of the macroscale connection from the $k^\textrm{th}$ region to the $i^\textrm{th}$ region, $R_{ik}$, we sum all of the individual strengths for all existing connections from neurons in the $k^\textrm{th}$ region to neurons in the $i^\textrm{th}$ region, such that $R_{ik}=\sum _{x \in Y_i} \sum _{y\in Y_k} A_{xy}$. As in the case of the full connectivity matrix, we see spatially closer regions are more strongly connected, agreeing with the intuition of the model.

### Complexity theory

In analyzing the potential evolutionary benefit of distance-dependent macroscale connectivity, we utilize the information-theoretic construct of neural complexity, which measures the degree to which a network integrates information among functionally separated groups of nodes. First, we briefly present the fundamental framework for neural complexity in terms of classical information theory. We consider a network of *n* neurons such that the activity of the full network at time *t* is described by $V(t)=\{v_i(t)|i=1, \dots , n\}$. The statistical independence of the network activity is conventionally measured by entropy,

5 $$\begin{aligned} H= - \sum _{i} p_i \log _{2} p_i, \end{aligned}$$

where $p_i$ is the probability of occurrence of the $i^\textrm{th}$ possible state of the network (Rieke et al. 1996). Entropy is therefore high if the network exhibits a large number of equally likely states and is minimal when the network exhibits only a single deterministic state. The total deviation from independence among a network of neurons can be measured using the difference between the sum of the individual neuron entropies and the entropy of the full network, which is known as the network *integration*,

6 $$\begin{aligned} I = \left( \sum _{i=1}^n H_i \right) - H, \end{aligned}$$

where $H_i$ is the entropy of the $i^\textrm{th}$ neuron. Note that $I=0$ only in the event of statistical independence among neurons, and integration increases with statistical dependencies. Comparing the integration of the full network and all possible subnetworks, the *neural complexity* is

7 $$\begin{aligned} C_n = \sum _{k=1}^{n-1} \left( (k/n)I - \langle I \rangle _k \right) , \end{aligned}$$

such that $\langle \cdot \rangle _k$ is the expectation over all $n!/(k!(n-k)!)$ subsystems of *k* neurons (Tononi et al. 1994). Neural complexity is high if the total integration of the system is high while simultaneously the integration over proper subsets of neurons is lower than would be expected from a linear increase with subset size. Thus, networks with high neural complexity demonstrate a high degree of differentiation in function among large modules of neurons while maintaining a high degree of interaction among specialized smaller sets of neurons.

When the activity of the full network can be well-described by statistically-stationary multivariate Gaussian dynamics, its entropy reduces to $H=0.5 \ln [(2 \pi e)^n|\text{ COV }(V)|]$. Here $\text{ COV }(V) = \overline{V^TV}$ is the covariance matrix expressed via averaging over a statistical ensemble, $\vert \cdot \vert$ denotes the determinant, and *H* is independent of *t* by stationarity (Rieke et al. 1996; Papoulis 1984). Note that subsets of neurons also follow multivariate Gaussian dynamics, and therefore their entropies can be described similarly. In this case, neural complexity, as defined in Eq. (7), is equivalent to

8 $$\begin{aligned} C_n = \sum _{k=1}^{n-1} 0.5 \left( \langle \ln |\text{ COV }(V) | \rangle _k - (k/n)\ln |\text{ COV }(V) |\right) , \end{aligned}$$

which is expressed in terms of the covariance matrix for the network dynamics and is consequently computable directly based on network simulation.

It is important to note that computing neural complexity for large networks using Eq. (7) is extremely computationally expensive and requires averaging over $2^n$ possible subsets; computing neural complexity using Eq. (8) is also challenging because it requires the computation of determinants unfeasible for sufficiently large networks. However, under the assumption of relatively weak inter-regional connection strength, it is possible to approximate neural complexity with only knowledge of the network connectivity. We refer to neural complexity computed in this manner as *structural complexity*, denoted $C_n^s$. Here, the macroscale network dynamics are approximated as a mulitvariate Ornstein-Uhlenbeck process, which yields dynamics analogous to a noisy Wilson-Cowan network model typically used to reflect the mean firing-rate activity for interacting clusters of neurons (Gardiner 2004; Wilson and Cowan 1973; Barranca et al. 2019).

In the weak inter-regional coupling limit, the neural complexity may be approximated to second-order, as described in detail in Ref. Barnett et al. (2009), by structural complexity

9 $$\begin{aligned} C_n^s \approx C^*_n+C^{**}_n, \end{aligned}$$

where the following quantities are given by sums over distinct indices

$$\begin{aligned} C^*_n=\frac{n+1}{48}\sum _{i \ne j}(A_{ij}^2+A_{ij}A_{ji}), \end{aligned}$$

$$\begin{aligned} C^{**}_n=&\frac{n+1}{96}\sum _{i \ne j \ne k}(3A_{ij}A_{jk}A_{ik}+ A_{ij}A_{jk}A_{ki} ) \nonumber \\&+ \frac{n+1}{24}\sum _{i \ne j}A_{ii}(A_{ij}^2+A_{ij}A_{ji}). \end{aligned}$$

It is important to emphasize that structural complexity only requires knowledge of the network connectivity matrix *A* and is inexpensive to compute even for large networks. As our model inter-regional connectivity is relatively weak for sufficiently large systems in Eq. (2) and each neuron receives weak external Poisson spike train input at a relatively fast rate, the microscale neurons operate in a nearly stationary regime (Cai et al. 2006, 2004; Barranca et al. 2014). Consequentially, as we will see in Sect. 3.3, the regional firing-rate dynamics are indeed approximately Gaussian and asynchronous across a broad region of parameterspace, facilitating the use of structural complexity to study the richness of the network topology.

## Results

### Structural complexity and inter-regional connectivity

**Fig. 2:**
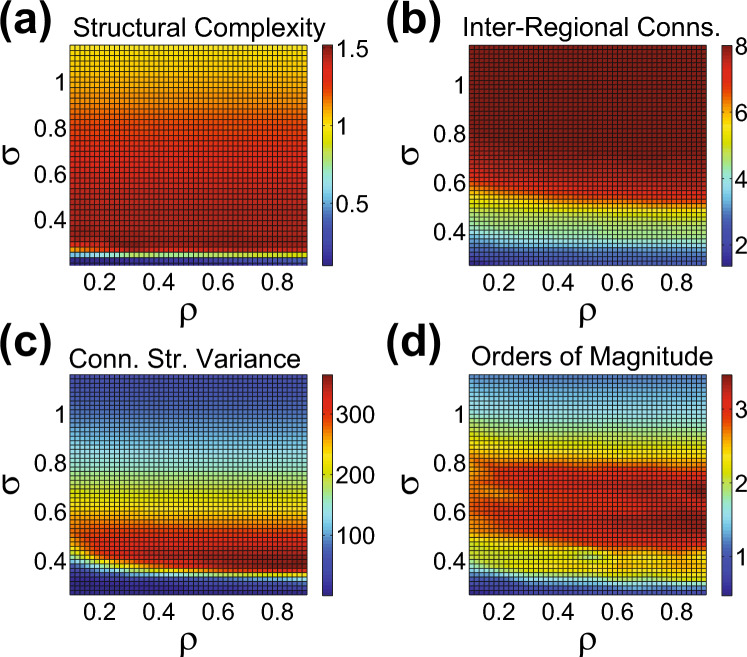
Network structure and distance-dependent connectivity. **a** Structural complexity of the cortical model plotted over the $(\rho , \sigma )$ parameterspace, determining, respectively, the density and distance over which inter-regional neuronal connections emerge. **b** Number of inter-regional incoming edges in the macroscale connectivity matrix *R*, averaged across all regions, over the $(\rho , \sigma )$ parameterspace. **c** Variance of the inter-regional connection-strengths in macroscale connectivity matrix *R* over the $(\rho , \sigma )$ parameterspace. **d** Maximum difference in the order of magnitude of inter-regional connection strengths over the $(\rho , \sigma )$ parameterspace. In (**b**), the $i^\textrm{th}$ region is said to receive an incoming edge from the $j^\textrm{th}$ region if at least one neuron from the $j^\textrm{th}$ region is connected to a neuron in the $i^\textrm{th}$ region (i.e., $R_{ij} \ne 0$). For each panel, there are $z=9$ cortical regions of $m=100$ neurons arranged on a square lattice, and the mean values over an ensemble of 10 realizations of the full network connectivity are depicted for each $(\rho , \sigma )$ parameter choice. The mean standard deviation across all realizations and parameter choices for (**a**–**d**), respectively, is 0.0306, 0.11, 7.27,  and 0.05

Using our multi-scale network model framework, we analyze the impact of distance-dependence in inter-regional connectivity on the structural complexity of the network. First, we do this by investigating the $(\rho ,\sigma )$ parameterspace, as described in Eq. (1), associated with the density and distance over which inter-regional connections emerge. In Fig. 2a, we plot the structural complexity of the macroscale connectivity matrix for each parameter choice. We observe a clear region of maximal structural complexity for intermediate $\sigma$ values, declining in the limit of both dense global connectivity and overly sparse localized connectivity. We also note that the density of inter-regional connections, controlled by $\rho$, has little impact on the structural complexity, with the distance over which connections generally occur, determined by $\sigma$, playing the more crucial role. In Fig. 2b, we depict the mean number of inter-regional incoming connections, affirming that the highest structural complexity is achieved when regional connections exist over moderate spatial scales, integrating a relatively small number of spatially localized functional modules.

To examine the particular macroscale network structure associated with high structural complexity, we examine the inter-regional connection strengths and their distribution over the $(\rho ,\sigma )$ parameterspace. We depict in Fig. 2c the variance in the inter-regional connection strengths in macroscale connectivity matrix *R* and observe a similar trend as in the case of structural complexity. The connection strengths are most variable for intermediate $\sigma$ values, exhibiting a relatively sharp decline in variability for excessive or insufficient distance-dependence. It is informative to point out that the parameter regime of maximal inter-regional connection strength variance exists well within the region of maximal structural complexity, with this high variability in connection strength potentially contributing to complex dynamics by facilitating a spatially localized modular structure among cortical regions.

In addition to highly heterogeneous inter-regional connection strengths, experimental studies have also indicated that these connection strengths span over several orders of magnitude (Markov et al. 2013a, b). To examine if this is also the case for our model, we plot in Fig. 2d the maximum difference in the order of magnitude of macroscale connection-strengths for each $(\rho , \sigma )$ parameter choice. We observe the largest span of inter-regional connection strengths for much of the parameter regime maximizing structural complexity. In the optimal case, the inter-regional connection-strengths span 3 to 4 orders of magnitude in our cortical model, showing strong agreement with experimental findings. Given structural complexity was derived under the assumption of relatively weak inter-regional connections, we note that the orders of magnitude of connection-strengths observed are primarily still small enough that the approximation is well justified, and the potential influence of larger inter-regional connections on complexity is further discussed in Sect. 3.3.

For robustness, we further note that all of the plots in Fig. 2 display values averaged over an ensemble of 10 network realizations for each parameter choice. For every panel, the standard deviation, reported in the figure caption, is extremely small, suggesting stability in these findings. Thus, we hypothesize that the heterogeneity in connection strengths experimentally observed in the cerebral cortex is a natural consequence of the distance-dependence in inter-regional connectivity, maximizing the network structural complexity when connections span intermediate spatial scales.

### Network dynamics and Gaussian statistics

**Fig. 3:**
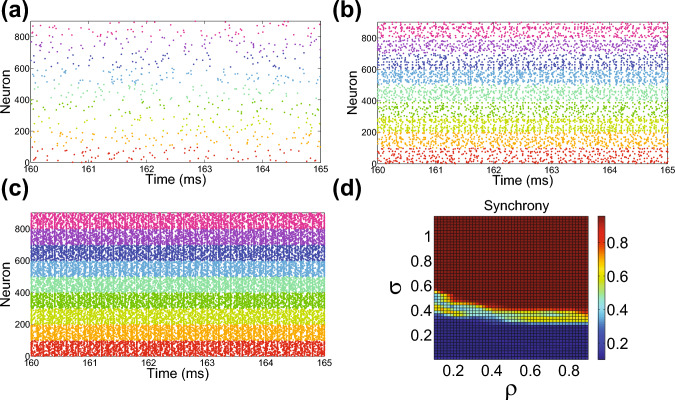
Distance-dependent connectivity and network dynamics. **a**–**c** Raster plots for spatial scales determined by $\sigma = 0.2, \sigma = 0.35,$ and $\sigma = 0.5$, respectively, with $\rho =0.9$ in each case. For each of the $z=9$ regions of $m=100$ neurons, the set of neuronal firing events is assigned a distinct color. Note that the network activity over a small subset of simulation time is depicted to make more visible the individual neuronal firing events. **d** Macroscale network synchrony measure, $\Omega$, plotted over the $(\rho , \sigma )$ parameterspace. In (**d**), the mean $\Omega$ values over an ensemble of 10 realizations of the full network connectivity are depicted for each $(\rho , \sigma )$ parameter choice, and the corresponding standard deviation is 0.01

While thus far we have primarily studied the model network structure in isolation from associated network dynamics, we now examine how the cortical network model activity is influenced by the degree to which inter-regional connection-strength is distance-dependent. Since the distance over which inter-regional connections manifest is primarily influenced by $\sigma$, we start by examining the evoked firing patterns across cortical regions for increasing values of $\sigma$ in Fig. 3a–c. Each raster plot exhibits a set of points corresponding to the indices of firing neurons as a function of the time of each respective firing event. To highlight the variation in dynamics between regions, we assign a unique color to neuronal firing events in each region.

As $\sigma$ increases, the dynamics gradually shift from relatively uncorrelated and incoherent to synchronous when the inter-regional connection density is sufficiently high. For intermediate distance-dependence, such as for $\sigma =0.35$, we note that there is a combination of synchronous firing among specific sets of neurons across regions and also uncorrelated activity among other sets of neurons. This simultaneously differentiated and integrated activity reflects the type of dynamics expected for a neuronal network with high complexity, which we will discuss further in Sect. 3.3.

We quantify more rigorously the degree to which the neurons in the various cortical regions synchronize using a macroscale synchrony measure. In the classical case of a single network of neurons, the synchrony measure characterizes fluctuations in the network-averaged voltage, $L(t) =(1/n) \sum _{i=1}^n v_i(t)$, relative to the average of the individual neuronal voltage fluctuations (Hansel and Sompolinsky 1992; Golomb and Rinzel 1993). The fluctuations in network activity are quantified by variance $\sigma _L^2= \langle \left[ L(t) \right] ^2 \rangle _t - \left[ \langle L(t) \rangle _t \right] ^2$, where $\langle \cdot \rangle _t$ denotes a time average. Similarly, activity fluctuations for the $i^\textrm{th}$ neuron are quantified by the variance, $\sigma _{v_i}^2,$ of $v_i$. Thus, the synchrony measure for a single network of *n* neurons is given by

10 $$\begin{aligned} \omega = \frac{\sigma _L^2}{(1/n) \sum _{i=1}^n \sigma _{v_i}^2}, \end{aligned}$$

where $0 \le \omega \le 1$ and larger $\omega$ values indicate higher synchrony. In the case of our particular model, instead of a single network of neurons, there are multiple cortical regions, such that each is a subnetwork of neurons that together compose the full network model. We therefore define an analogous *macroscale synchrony measure*, $\Omega$, to quantify the synchrony among the various cortical regions. We consider each subnetwork to be a single node in the macroscale network and compute the average voltage over each region, denoting the average voltage in the $i^\textrm{th}$ region by $L_i(t)$. Replacing $v_i$ with $L_i$ in Eq. (10) and averaging over the *z* cortical regions gives the macroscale network synchrony measure,

11 $$\begin{aligned} \Omega = \frac{\sigma _L^2}{(1/z) \sum _{i=1}^z \sigma _{L_i}^2}, \end{aligned}$$

which exhibits similar characteristics as the classical single-network synchrony measure but on a more coarse-grained level. In Fig. 3d, we compute $\Omega$ over the $(\rho , \sigma )$ parameterspace averaged over an ensemble of realizations of the full network connectivity. We generally observe increasing synchrony with $\sigma$, and a particularly rapid transition in $\Omega$ for intermediate levels of distance-dependence. The dynamics in this intermediate parameter regime well correspond to those we would expect for a highly complex network, exhibiting a rich mix of correlated and uncorrelated dynamics among cortical regions. Moreover, we have observed that the same trends hold for the fraction of variance explained by the first three principal components of the network-averaged voltage, demonstrating higher redundancy for larger $\sigma$ values (Pearson 1901; Abdi and Williams 2010).

**Fig. 4:**
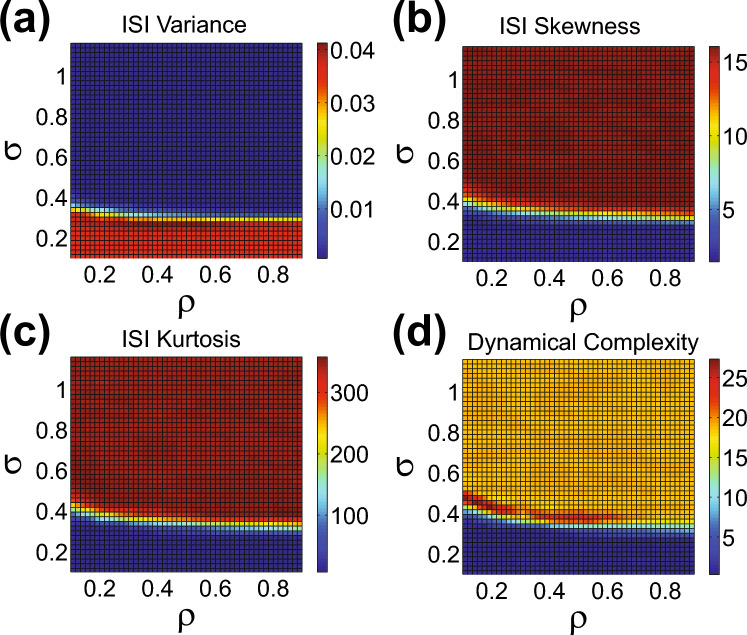
Gaussianity and complexity of network dynamics. **a**–**c** Variance, skewness, and kurtosis of the interspike interval (ISI) distribution, respectively, plotted over the $(\rho , \sigma )$ parameter space. Note that the distribution includes the ISIs over all firing events of all neurons in the full network model with $z=9$ regions of $m=100$ neurons. **d** Dynamical complexity of the network plotted over the $(\rho , \sigma )$ parameter space. In (**a**–**d**), the mean values over an ensemble of 10 realizations of the full network connectivity are depicted for each $(\rho , \sigma )$ parameter choice. The mean standard deviation across all realizations and parameter choices for (**a**–**d**), respectively, is 0.0012, 0.12, 4.08,  and 0.43

The dynamics of the cortical network model can also be well understood in terms of the statistics of the network interspike intervals (ISIs), which each denote the amount of time between two subsequent firing events of a particular neuron. We investigate the variability in the ISIs over all neuronal firing events in the full network as a function of the inter-regional connectivity parameters $\rho$ and $\sigma$. In Fig. 4a–c, we plot the variance, skewness, and kurtosis of the ISI distribution, respectively. The skewness, $\langle (\textrm{ISI} - \langle \textrm{ISI}\rangle )^3\rangle /\sigma ^3_\textrm{ISI}$, measures the asymmetry of the ISI distribution and the kurtosis, $\langle (\textrm{ISI} - \langle \textrm{ISI}\rangle )^4\rangle /\sigma ^4_\textrm{ISI}$, measure the tailedness of the ISI distribution. Both can be used to characterize how closely a given distribution resembles a Gaussian, which has 0 skewness and 3 kurtosis in this framework.

We observe in Fig. 4a a small region of maximal ISI variance for intermediate $\sigma$ values, with high $\sigma$ yielding the lowest ISI variance as the full network becomes more synchronized and neurons fire at approximately the same rate. This highly variable ISI distribution for moderate $\sigma$ is a good indicator of high complexity in network dynamics, and it exists well within the region of maximal structural complexity discussed in Sect. 3.1. As in the case of structural complexity, the statistics primarily depend on $\sigma$ and exhibit stability over realizations of the network connectivity.

With respect to the Gaussianity of the ISI distribution, we observe relatively low skewness and kurtosis in Fig. 4b, c for both low and intermediate values of $\sigma$. In this region of parameterspace, the network dynamics are approximately Gaussian. For yet higher values of $\sigma$, the distribution exhibits a rapid increase in both skewness and kurtosis, in which case the network is primarily synchronous.

In the high $\sigma$ limit, distant neurons and nearby neurons are nearly just as likely to be connected, effectively diminishing the spatial dependence and heterogeneity in the inter-regional network coupling. With the connection strengths defined in Eq. (2), as the full network becomes more connected, the strength of each connection decreases, conserving the expected drive of each neuron. In this case, the effective input from neighboring neurons may be treated as approximately constant in time and uniform throughout each regional network, and therefore the full network dynamics become quite synchronous, especially if the external drive into each region is sufficiently similar. At the same time, the network also enters a high-firing state due to the nearly constant bombardment of inputs from neighboring neurons. Even though the Gaussian approximation for the dynamics does not hold in this high $\sigma$ regime, since the network dynamics are so clearly synchronous and correlated, it is still reasonable to assume low complexity as the regional activity is significantly more integrated than differentiated.

Similarly, for low $\sigma$, in which case there are very few, if any, inter-regional connections, the lack of communication among neurons in different regions and low correlation in even nearby regional activity forces the full network into a more separated than integrated state. Since the asynchronous network dynamics are approximately Gaussian for low $\sigma$, we may view the structural complexity metric as applicable in this appropriate dynamical regime. However, only for intermediate $\sigma$ values do we additionally observe a diverse ensemble of correlated and uncorrelated dynamics across cortical regions, which is necessary for balanced integration and differentiation of information.

Alternatively, we note that in the case when the spiking-neuron dynamics are not Gaussian, we could view the structural complexity results as applicable to the rate-model formulation of our network dynamics under the same assumptions on the inter-regional network structure. One could reasonably analyze the network dynamics of each region using a rate-model under the assumption that the dynamics are coarse-grained over firing events in each cortical region, freeing the notion from a particular choice of microscale model dynamics. Nevertheless, we view the dynamics in the regime of interest as close enough for direct application of the Gaussianity assumption in computing the structural complexity of our network model, and we expect that complex neuronal dynamics may be intimately linked to Gaussianity in ISI structure in general. In fact, approximately Gaussian dynamics appear to be a ubiquitous operating regime in the brain, facilitating particularly high capacity for information encoding (Barranca et al. 2014; Wilson and Cowan 1973; Nunez and Srinivasan 2006; McIntosh and Gonzalez-Lima 1994).

### Dynamical complexity

We conclude by introducing a coarse-grained notion of *dynamical complexity*, which relies only on measurements of network dynamics and is particularly inexpensive to compute given the multi-scale nature of our model. Upon measuring network activity, the most basic option for determining the dynamical complexity would be to directly apply the definition given by Eq. (8) over the entire neuronal network. However, this would require computing the covariance matrix over all $2^n$ subsets of individual neuronal activity for a network of *n* neurons. For large networks, this would typically not be a computationally feasible approach. Instead, we utilize our network model at the regional level to define a more computationally efficient framework for measuring the complexity of network dynamics. Rather than considering each individual neuron to be a node in the full network, we consider each region to be a node in the macroscale network. In this case, the average voltage dynamics over the $i^\textrm{th}$ region, $L_i(t)$, constitutes the dynamics of the $i^\textrm{th}$ node, and therefore only *z* nodes are necessary to consider for a network of *z* regions. In this way, we define *dynamical complexity* as

12 $$\begin{aligned} C_z^d = \sum _{k=1}^{z-1} 0.5 \left( \langle \ln |\text{ COV }(L^F) | \rangle _k - (k/z)\ln |\text{ COV }(L^F) |\right) , \end{aligned}$$

in terms of the covariance matrix for the regional network activity described by $L^F(t)=\{L_i(t)|i=1, \dots , z\}$. Therefore, only $2^z$ rather than $2^{zm}$ possible subsets need to be considered, offering particularly large computational savings for the realistic case in which the number of neurons contained in each region is much larger than the total number of regions (i.e., $m \gg z$ ).

We consider how the dynamical complexity is influenced by distance-dependence in the inter-regional connectivity in Fig. 4d as we vary parameters $\rho$ and $\sigma$. We observe that the dynamical complexity is maximized for connectivity over intermediate spatial scales. For low $\sigma$, in the regime of relatively uncorrelated dynamics, the dynamical complexity is particularly low, and for high $\sigma$, in the relatively synchronous and correlated regime, the dynamical complexity is moderate. While we do observe a maximum dynamical complexity that indeed exists within the regime of maximal structural complexity and the two types of complexity exhibit a similar dependence on the macroscale network structure, the two notions do not precisely correspond, as seen through comparison of Figs. 2a and 4d.

Further study would be necessary to pinpoint the exact differences in interpretation of the two notions of complexity, but both previous work and Eq. (9) demonstrate that structural complexity primarily selects for various graphical constructs, such as bidirectional connectivity and cyclic graph motifs, whereas dynamical complexity directly takes into account the model activity rather than the associated network structure (Barnett et al. 2009). As the network dynamics become more correlated, it is expected that higher-order motif structures ignored in our notion of structural complexity may become more important. Though the assumption of weak inter-regional correlations in neuronal dynamics can be viewed as a limitation for structural complexity, there is compelling evidence that highly asynchronous and irregular activity is prominent in both spontaneous and evoked conditions (London et al. 2010; Xue et al. 2014a; Haider et al. 2006; Tan and Wehr 2009; Runyan et al. 2010).

On the other hand, considering detailed neuronal network structure is difficult to measure for large systems (Barranca 2023), the calculation of dynamical complexity may be more feasible in practice for data-driven studies because it does not require knowledge of network connectivity. Since the network model dynamics are generally only Gaussian with weak inter-regional connectivity for low to moderate $\sigma$, it may be reasonable to exclude the high $\sigma$ limit from this particular analysis, especially since the dynamics are clearly synchronous and not complex in this regime. The assumption of stationarity, utilized for both dynamical and structural complexity, may not be appropriate for certain evoked conditions, particularly with fast or unpredictable stimuli, but could reasonably be satisfied under certain spontaneous conditions, sleep, or anesthesia. However, for non-stationary data, recent work analyzed a complexity time-series for various brain regions computed via weighted permutation entropy applied to functional magnetic resonance imaging signals, underlining a default high complexity state accompanied by transient complexity drops potentially indicative of functional subsystems (Krohn et al. 2023). Similarly, the degree to which the two complexity measures might still succeed in evoked conditions by applying windowing or sufficient averaging over many stimuli remains a subject of further inquiry. In either case, the notions of structural and dynamical complexity do indeed underline how distance-dependent inter-regional connectivity potentially plays an important role in facilitating rich neuronal computations characterized by balanced integration and differentiation when inter-regional connectivity spans moderate spatial scales.

For robustness, we have also compared the results presented in this work to those generated using alternative network models. For example, by incorporating delayed synaptic communication, the only key difference we have observed is that the onset of synchrony often occurs for larger $\sigma$ values given sufficient delay, which is to be expected considering delayed transmissions increase the amount of time necessary for neuronal activity to enter stable limit cycles (Koch 1999; Gregoriou et al. 2009). Similarly, for the case in which $20 \%$ of local connections are inhibitory, as observed in several brain regions (Cai et al. 2005; Wu 2010; Xue et al. 2014b; Barranca et al. 2022), the results are comparable as long as the inhibition strength is relatively low and does not dominate the average excitatory input; for larger inhibition in which the network may enter a different dynamical regime, however, further investigation into the complexity of the dynamics is warranted. For larger networks, with $z=16$ or $z=25$ regions with an analogous square lattice geometry, we have verified a similar role of distance-dependence in our network model and observed an analogous optimal $\sigma$ range, with higher structural and dynamical complexity values generally achieved for networks containing more regions.

## Discussion

Using a multi-scale mechanistic model of the cerebral cortex with nonlinear spiking dynamics at the individual neuron level and inter-regional connection strength decaying exponentially with distance, we have studied the role of distance-dependent connectivity in determining the complexity of network structure and dynamics. For the case in which inter-regional connections span over a moderate spatial scale, we found maximal heterogeneity in inter-regional connection strengths. As a natural consequence, we observed that the inter-regional connection strengths range over several orders of magnitude, agreeing well with previous experimental findings (Markov et al. 2013a, b). Formulating macroscale structural and dynamical notions of complexity, we observed that the model parameter choices giving rise to maximal inter-regional connection strength variability also facilitated optimal complexity, with balanced integration and differentiation of regional activity. In the regime of high complexity, neurons in differing regions demonstrated a combination of synchrony and uncorrelated activity, with approximately Gaussian ISI statistics. We hypothesize that highly complex dynamics are facilitated by modularity and distance-dependence in neuronal connectivity, which have likely been selected by evolution for their capacity to efficiently process information.

The coarse-grained framework of dynamical and structural complexity formulated in this work is well suited for multi-scale networks and particularly offers a computationally tractable means of analysis for large neuronal networks where it may be of interest to examine the link between dynamics at the level of individual neurons, the network connectivity structure, and the resultant neural complexity. Considering many data sets of interest involve a small number of brain regions or functional modules relative to total neurons, it is unwieldy to apply notions of complexity considering all subsets of individual neurons, rather than the relatively small number of regions considered in the coarse-grained framework. Nevertheless, it would be interesting to compare the results gleaned by the coarse-grained and full-network complexities given sufficient computing resources. While the approximations of neural complexity introduced previously for single-scale systems with a relatively small number of nodes (Barnett et al. 2009) indeed provide important insights into the network topological underpinnings of the originally formulated neural complexity (Tononi et al. 1994), our work extends their application to larger-scale nonlinear systems with distinct functional modules and helps to push the boundary of the typical assumptions of strict Gaussianity, linearity, and stationarity, which still marks a frontier in the field. Additionally, though we have specifically chosen to study the role of distance-dependence using the framework of a multi-scale network with complexity coarse-grained over subnetworks, the developed methodology is well suited for characterizing the impact of other common neuronal network structures, such as receptive field types and motifs, on the complexity of network structure and dynamics (Wiesel 1960; Hubel and Wiesel 1960).

Broadly construed, abnormalities in the complexity of brain dynamics have been implicated in a number of neurological disorders (Lau et al. 2022), including schizophrenia, autism, and depression (Garrity et al. 2007; Whitfield-Gabrieli et al. 2009). In the cases of autism (Kang et al. 2019) and Alzheimer’s disease (Fan et al. 2018), for example, most evidence points to an association between reduced complexity and increasing severity of the disease. An imbalance of synchronous activity has similarly been associated with epilepsy, with one theoretical study linking epilepsy with excessive long-distance connections (Netoff et al. 2004). At the same time, as individuals age, complexity generally increases into adulthood and then decreases into old age (Lippé et al. 2009), which in part may be due to pruning of long-range connections beyond adulthood (Thatcher et al. 2008; Tomasi and Volkow 2012), and excessively diminished long-range connectivity has been observed in Alzheimer’s disease (Liu et al. 2014).

From the results of this work, we conjecture that the diminishing strength of inter-regional connections with distance may be a fundamental mechanism for preventing excessive synchronization from manifesting across the brain. While the irregular and asynchronous neuronal activity stemming from balanced excitatory and inhibitory inputs has been found to well characterize neurotypical awake brain activity (London et al. 2010; Haider et al. 2006; Vreeswijk and Sompolinsky 1996), it would be reasonable to assume that additional structural constraints may also help to naturally keep activity in cortical regions within an acceptable dynamical regime (Shadlen and Newsome 1994; Isaacson and Scanziani 2011; Barranca et al. 2019). We expect that maximal complexity is inherent in brain networks under constraints on a combination of features, such as activity levels, energy expenditures, and information processing time, and complexity may further provide a useful framework for understanding abnormal brain states both at regional, as in this work, as well as individual neuron levels.

## Data Availability

Data will be made available upon reasonable request.
